# Treatment approaches for managing patients with hematological malignancies in the time of COVID-19 pandemic

**DOI:** 10.3906/sag-2101-276

**Published:** 2021-03-11

**Authors:** Güldane CENGİZ SEVAL, Pervin TOPÇUOĞLU, Taner DEMİRER

**Affiliations:** Department of Hematology, Faculty of Medicine, Ankara University, Ankara, Turkey

**Keywords:** COVID-19, SARS-CoV-2, hematological malignancies

## Abstract

**Background/aim:**

The COVID-19 pandemic is a unique challenge to the care of patients with hematological malignancies. We aim to provide supportive guidance to clinicians making individual patients decisions during the COVID-19 pandemic, in particular during periods that access to healthcare resources may be limited.

**Conclusion:**

This review also provides recommendations, which are convenient in evaluating indications for therapy, reducing therapy-associated immunosuppression, and reducing healthcare utilization in patients with specific hematological malignancies in the COVID-19 era. Specific decisions regarding treatment of hematological malignancies will need to be individualized, based on disease risk, risk of immunosuppression, rates of community transmission of SARS-CoV-2, and available local healthcare resources.

## 1. Introduction

At the end of 2019, Wuhan city, the capital of Hubei province in China, became a center of an outbreak of pneumonia of unknown cause. By January 2020, Chinese scientists had identified a novel coronavirus of zoonotic origin, severe acute respiratory syndrome coronavirus-2 (SARS-CoV-2; previously known as 2019-nCoV), from these patients with virus-infected pneumonia. This infection was named coronavirus disease 2019 (COVID-19) by the WHO and is now spreading worldwide [1]. As widespread community transmission becomes likely, it has posed an unprecedented health emergency. As of 31 December 2020; 85.1 million cases of COVID-19 have been confirmed resulting in 1.84 million deaths worldwide^1^. Avoiding exposure by adhering to recommended hygiene procedures, isolation of SARS-CoV-2-infected individuals, and social distancing especially for risk groups are currently the main prevention strategies utilized in most countries [2].

Patients with hematological malignancies are given cytotoxic therapies, usually administered with curative intent and management of these patients during the current pandemic of COVID-19 is challenging [3]. Hematologic cancer patients require special attention and decision making regarding therapy during the COVID-19 pandemic and needs specialized consideration. It is important to note that, the quality of care for these patients were also affected and strategies are required to deliver the best care during this pandemic.

Herein, we reviewed some of recommendations to help with the management of patients with hematologic malignancies as well as the evidence when available. Although data regarding patients with hematological malignancies are scarce, the considerations for care modification consist of oral and/or outpatient choices, protocols that decrease the risk of cytopenias and postponement of treatment if possible. Clinical study enrollment is significantly limited, and the risk-benefit ratio of experimental treatments and their required logistics must be reconsidered. In the present paper, we intended to delineate the best practices for myeloid and lymphoid neoplasms as well as multiple myeloma during the current COVID-19 pandemic.

## 2. General considerations and supportive care

Whether the risk of contamination with SARS-CoV-2 is higher in the inpatient or outpatient setting for patients with hematological malignancies depends on COVID-19 incidence in the local community [4]. Self-isolation may enable patients to delay or avoid COVID-19 and this may be of critical importance following chemotherapy. The high risk of nosocomial spread of SARS-CoV-2 in hospital settings is known and also concerns about overwhelming inpatient capacity limitations are well known [5].

In our center, we have focused on patient and caregiver education about the importance of social distancing, hand hygiene, and masking. Patients are confined to one caregiver and no visitors are allowed in to inpatient unit. In addition, entrance into our center has been closed to a single point, at which all patients, staff, and caregivers are screened; those with symptoms concerning for COVID-19 are instructed to COVID-19 outpatient clinic for testing. Patients with known respiratory symptoms and fever are referred to the emergency room. Outpatient clinics started to work with telephone appointments or video-conferencing arrangements wherever these were felt to be acceptable.

Routine testing of asymptomatic patients for SARS-CoV-2 is particularly challenging. Most experts recommend screening cancer patients for SARS-CoV-2 and the test results should be negative before initiation of the chemotherapy regardless of whether fever or respiratory symptoms are present [2]. If an immediate treatment is needed, standard therapy should be administered in a COVID-19 positive environment. However, testing capacity and availability of these tests are country dependent.

Most experts recommended increased use of granulocyte colony stimulating factor (G-CSF) and antibiotic prophylaxis routinely to reduce admissions for febrile neutropenia (FEN) in the current situation [4]. There are also some theoretical concerns about the exacerbation of SARS-CoV-2 respiratory effect with filgrastim, but as of yet there is no contraindication to G-CSF support in patients anticipated to become FEN. Bacterial secondary infection can complicate viral infections, a situation well known in influenza, and also plausible for SARS-CoV-2. Vaccination against Streptococcus pneumonia should be recommended to immunocompromised patients but this needs to be worth investigating in clinical trials [5]. In our center, we recommend all lymphoma and myeloma patients receive routine influenza and pneumococcal vaccination.

There is no shown instance of SARS-CoV-2 transmission through blood products, but most transfusion societies call for conservative transfusions policies in strict adherence to evidence-based guidelines for patient’s blood management in the presence of decreased donor availability [5]. For patients without symptomatic anemia or bleeding complications, consider decreasing the hemoglobin and platelet thresholds to 7 g/dL and 10 × 10^9^/μL, respectively. According to the American Society of Hematology (ASH) recommendations, antifibrinolytics can be given for patients requiring frequent platelet transfusion and/or platelet-transfussion-refractory patients^2^.

Early discussions about goals of care are important in this pandemic era which may lead to increased complications and comorbidities among patients with cancer, who are elderly or have associated chronic disabling diseases [4].

## 3. Myeloid neoplasms

### 3.1. Acute myeloid leukemia

As 50%–75% of patients with acute leukemia are febrile at diagnosis, they may encounter to the problem of missed or delayed diagnosis across pandemic period (5). In addition, most patients may suffer from postponement of chemotherapy, due to a limited isolation beds and blood products. Delay in chemotherapy initiation may unfavorably affect prognosis, especially in young (<60 years-old) leukemia patients with favorable- or intermediate-risk disease [5].

All the newly diagnosed patients with acute myeloid leukemia (AML) should be screened for SARS-CoV-2 regardless of symptoms, including baseline CT of the chest prior to induction as well as consolidation treatment if indicated. The overall treatment of those patients should not change during the COVID-19 pandemic with a few caveats [6].

#### 3.1.1. Induction therapy

While newly diagnosed AML is considered treatment of emergency in most cases, intensive induction chemotherapy should still be preferred for eligible patients with 7+3 (cytarabine plus anthracycline) or a similar one. According to recommendations of Seattle group, lower-intensity therapies such as venetoclax with hypomethylating agents (HMA/VEN) or low-dose cytarabine are given to the patients for administering outpatient induction [4]. However, Paul and his colleagues from MD Anderson Cancer Center (MDCC) generally recommend these therapies primarily for patients aged >60 years or those deemed unfit for intensive chemotherapy [6]. Although the regimens have not been compared with high-intensity therapy and are not typically suggested for patients aged <60 years who are otherwise fit for intensive therapy, they may be considered in areas with high incidence of COVID-19 cases to minimize transfusion and utilization of inpatient beds [6]. It is important to note that there is no evidence that HMA/VEN is less toxic than standard induction therapy and patients experience long phases of aplasia, requiring close follow-up with routine blood counts. MDCC group recommended a bone marrow evaluation around day 14–21 during the first cycle to reduce the period of myelosuppression with VEN combinations. If there is no morphological evidence of leukemia, VEN may be withdrawn and administration of G-SCF can be considered to fasten neutrophil recovery [6].

Based on our knowledge, AML induction therapy does not primarily influence the lymphocytic cells and patients are more vulnerable to bacterial and fungal rather than viral infections. Prophylactic antimicrobials should generally include levofloxacin, posaconazole, and acyclovir in the prolonged neutropenia setting.

#### 3.1.2. Consolidation therapy

AML patients in complete remission (CR) undergoing consolidation/post-remission therapy should receive outpatient care when possible. European Hematology Association (EHA) recommends only postponing the consolidation chemotherapy until the virus is cleared in COVID-19 positive patients. With regard to the dosage, intermediate-dose cytarabine (1.5 g/m^2^) is recommended to COVID-19 positive but also to COVID-19 negative AML patients due to the lack of overall survival (OS) benefit with the higher dose of 3 g/m^2^. In this regard, the day 1/2/3 schedule should be preferred over the 1/3/5 schedule with a view to decreasing the number of cycles to three instead of four. The addition of GCSF with this condensed cytarabine schedule has been reported to shorten the time to neutrophil recovery, risk of infection, duration of hospitalization and platelet transfusion requirement^3^.

Consolidative allogeneic hematopoietic stem cell transplantation (AHSCT) is recommended for patients with intermediate or high-risk genomic characteristics, but availability is currently limited [4]. In general, however, if a transplant candidate is diagnosed with COVID-19 a postponement of at least three months is advisable, in accordance with European Society for Blood and Marrow Transplantation (EBMT) and European Centre for Disease Prevention and Control (ECDC) recommendations [2]. However, this is not always possible due to the risk of progression of the underlying leukemia. Therefore, in patients with high-risk disease, AHSCT should be deferred until the patient is asymptomatic and has two negative SARS-CoV-2 PCR swabs taken at least 24-h apart. The potential contribution of anti-SARS-CoV-2 antibody detection is presently unclear but should be done if available. Cryopreservation of donor cells prior to the start of conditioning should be preferred before proceeding to transplant, which may require an extra cycle of consolidation therapy^4^.

#### 3.1.3. Maintenance therapy

Maintenance therapy should be offered particularly for patients who may be unable to receive their intended intensive consolidation courses. Based on the results of the QUAZAR maintenance trial, oral azacitidine improved relapse-free survival (RFS) and OS in patients being ≥55 years who had previously achieved CR with intensive induction therapy [7]. Given the deferral in AHSCT during COVID-19 pandemic, maintenance azacitidine ± VEN (NCT0406226) should be suggested after patients have completed consolidation therapy while awaiting AHSCT as an alternative post-consolidation approach (for 1–2 years) in order to maintain remission [6].

#### 3.1.4. Treatment of relapsed/refractory AML

Many clinical trials have intermitted enrollments, so standard salvage regimens are recommended if unable to access a clinical trial. Intensive re-induction for salvage are still recommended, but the potential benefit must be weighed against the hardship for patients admitted for prolonged hospital stay and the shortage of blood products. ASH recommended for patients without proliferative disease or significant transfusion dependence to consider postponing the chemotherapy^5^. Some centers are resuming to prioritize clinical trials for this patient population. Also patients should undergo AHSCT if clinically indicated and safe [7,8].

Genomic testing should be done at the time of relapse to pinpoint the efficacy of approved targeted agents such as for Isocitrate dehydrogenase-1 (IDH-1), IDH-2, or farnesyl transferase-3 (FLT-3) mutations [6]. Utilizing an outpatient, lower-intensity regimen alone or in combination with targeted therapies whenever available should be preferred. IDH inhibitors, ivosidenib or enasidenib may be effective in AML with IDH1 or IDH2 mutations, respectively. Both inhibitors play a role as differentiating agents with the occurrence, in 10%–20% of patients, of a differentiation syndrome, which requires immediate corticosteroid administration and intensive care support [5]. There is no publication in the literature about the risk of severe respiratory failure in patients received these agents and infected by SARS-CoV-2.

### 3.2. Acute promyelocytic leukemia

Patients with newly diagnosed low-risk acute promyelocytic leukemia (APL) should be treated with the combination of all-trans retinoic acid (ATRA) and arsenic trioxide (ATO) as per standard protocol. High-risk APL patients should be given cytoreductive chemotherapy in addition to ATRA and ATO as per standard protocol [6].

APL patients at high risk of differentiation syndrome can also be treated with prophylactic dexamethasone but the depth of lymphopenia is of unknown risk in relation to COVID-19.

### 3.3. Myelodisplastic syndrome

Myelodisplastic syndrome (MDS) was diagnosed often in elderly population with a median age of 68 years at diagnosis. Elderly patients are more likely to have comorbidities compromising the immune system and this cause to develop high risk of severe COVID-19. But also there are no published data yet indicating that patients with MDS or related conditions are more prone to COVID-19 than patients with normal bone marrow function. However, in the presence of the neutropenia and functional neutrophil defects among patients with MDS, there has been an increased risk of bacterial and fungal infections to a much greater than the risk of viral infections.

For patients with a newly diagnosed MDS during the COVID-19 pandemic, selection of therapy should depend on a risk-adapted approach using the revised international prognostic scoring system (IPSS) and following standard guidelines [6].

For patients with lower-risk MDS (IPSS-risk(R) score <3.5), the clinicians should consider to reduce transfusions and improve quality of life (QoL). In patients who are transfusion-independent, initiation of therapy should be postponed to minimize the need for clinic visits and viral exposure. Therapy with erythropoietin-stimulating agents (ESA) may result in a net decrease of transfusion needs [6].

For patients with higher-risk MDS (IPSS-R score ≥3.5) treatment should be initiated with HMA without delay, and dose adjustment. HMAs should be continued to prevent the progression of disease to AML if they are already responding [6]. Despite of the potential risk of aggravating cytopenias with HMA during the first two cycles of treatment, the response to HMA in ultimate recovery of cytopenias and favorable impact on survival offsets this risk in patients with higher-risk MDS [6].

Intensive therapy can be considered for patients with higher-risk MDS in whom HMA have failed in spite of the higher risk of prolonged myelosuppression, infections, and the potential increased mortality in the time of COVID-19 [5,6]. The management will be challenging given the absence of approved therapies in this population and clinical trials may represent the only treatment option. AHSCT may still be feasible but given the potential expected delays of AHSCT during the COVID-19 pandemic, bridging therapy may be needed. Treatment with low doses of cytotoxic agents such as cladribine or clofarabine with low-dose cytarabine should only be preferred in selected patients with higher-risk MDS and normal karyotype, provided COVID-19 swab testing is negative [5,6].

### 3.4. Myeloproliferative neoplasms

Until now, there is too limited data about patients with myeloproliferative neoplasm (MPN) who were infected by SARS-CoV-2. It is clear that nonimmunosuppressive drugs including low-dose aspirin, phelobotomy, hydroxyurea (HU), anagrelide, and interferons (IFN) should be continued since they reduce the risk of short-term complications such as thrombosis, bleeding, disease-related symptoms, and splenomegaly [4]. Therefore, ASH is not recommending any adjustments to the aforementioned therapies in someone without/without COVID-19 but suggesting to initiate cytoreductive treatments weighing the risk and benefits for patients with polycythemia vera (PV) and essential thrombocythemia (ET)^6^.

Phlebotomy is initiated for newly diagnosed patients with PV, but the other issue is that clustering large groups into waiting areas for phlebotomy has the potential risk to facilitate the spread of the COVID-19. Therefore, it’s recommended to consider extending the interval between blood draws to monitor counts in order to defer outpatient visits. JAK inhibitors (JAKi) have also been commonly used as part of effective treatment for MPN. Initiation of JAKi (ruxolitinib) may result in worsening anemia early in therapy and may cause atypical infections. The abrupt discontinuation of JAKi in successfully controlled patients with MPN may lead to weakness, progressive splenomegaly, or infrequently cytokine storm, and altogether these can exacerbate the clinics of patients who are infected with SARS-CoV-2^5^. On the other hand, the global phase III RUXCOVID clinical trial evaluating ruxolitinib in combination with standard care for the treatment of cytokine storm triggered by COVID-19 has been initiated (NCT04362137). For JAKi responder patients with MPN, treatment should be continued but if JAKi therapy is not considered, it is recommended to postpone until after the peak of the COVID-19 pandemic has declined.

AHSCT is currently suggested to patients with high-risk myelofibrosis (MF) according to Dynamic international prognostic scoring system (DIPSS)-Plus or equivalent after carefully weigh the risks and benefits. However, based on the ASH recommendations, work up for AHSCT can proceed but transplant would be deferred until the risk of COVID-19 has subsided^5^.

### 3.5. Chronic myeloid leukemia

Chronic myeloid leukemia (CML) treatment depends on the administration of continuous BCR-ABL tyrosine kinase inhibitors (TKIs). To the best of our knowledge, patients with CML are not at a high risk of infection due to underlying disease or BCR-ABL TKIs. Therefore, in newly diagnosed CML, delayed initiation of TKI therapy is not recommended because uncontrolled leukocytosis might aggravate lung injury and gas exchanges in the event of COVID-19 infection. CML progression to advanced-phases may be even more important risk due to postponed initiation of TKI.

For newly diagnosed patients, the side effect profiles of each TKIs should be evaluated in the presence of symptoms consistent with SARS-CoV-2, such as lung damage, cardiomyopathy, cardiac arrest, diarrhea, and thrombotic events. All these may be worsened with TKIs that can cause similar toxicities and dismal outcomes of COVID-19 [6]. Therefore, newly diagnosed patients with CML should be tested with nasopharyngeal swab. If they have any active infection, TKIs should be held to decrease additional lung stress during infection and recovery [6]. There is no data suggesting that any of the TKI currently approved for first line therapy led to a greater or lower risk of COVID-19 or worse outcome. Some experts advised to continue TKI treatment in the presence of nonsevere confirmed COVID-19; in case of severe course of COVID-19, TKI interruption should be handled case-to-case basis^7^.

For patients with CML on TKIs, prophylactic interruptions are not recommended because such an approach may result in loss of response and relapse/progression. According to EHA recommendations; patients with CML who discontinued TKI for less than one year and who do not have access to regular monitoring of CBC counts and BCR-ABL transcripts in time of COVID-19 pandemic are suggested to discuss with their doctors to restart TKI treatment^6^. Furthermore, patients who have attempted treatment-free remission (TFR) are in need of more frequent monitoring, as often as monthly and require continuing if at all possible. Initiation of TFR attempts may be postponed during the COVID-19 pandemic due to the increased requirement for monitoring that may not be applicable^6^.

Patients with accelerated-phase CML responding well to TKI may continue with proper monitoring. Transformation to accelerated phase while on TKI can be treated with a different TKI and close monitoring. Blast phase CML should be treated with intensive chemotherapy in combination with TKI if local conditions allow for close monitoring, adequate supportive care (blood product transfusion, management of possible infections) and carefully weigh the risks and benefits^6^.

It is important to note that, attention should be given to concomitant medications, which may interfere with TKI metabolism in the face of a known COVID-19. Drug interactions that may cause QT prolongation should be taken into consideration when potential SARS-CoV-2 infection directed therapies given with TKI treatment and, therefore, require proper EKG monitoring^6^.

## 4. Lymphoid neoplasms

### 4.1. Acute lymphoblastic leukemia

Treating patients with acute lymphoblastic leukemia (ALL) in the time of COVID-19 pandemic can be particularly challenging. The recent developments of ALL treatments are based on less myelosupressive regimens incorporating the CD3–CD19 bispecific antibody, blinatumomab, and the anti-CD22 conjugated antibody, inotuzumab ozogamicin [6]. These are advanced therapy choices and overall safety and efficacy were shown in numerous clinical trials, especially in elderly patients.

Sars-CoV-2 PCR testing should be performed regardless of symptoms, including baseline CT of the chest due to the potential for false-negative PCR from nasopharyngeal swab, prior to induction as well as consolidation treatment if indicated [6]. If the PCR testing is positive, we should consider deferring treatment by 10–14 days, except intrathecal therapies for CNS involvement. If the patient is negative for SARS-CoV-2, consider repeating the test after 24 h if there is high clinical suspicion [9].

#### 4.1.1. Ph-negative ALL

In ALL, one important question is the use of glucocorticoids, as they still remain major components of treatment protocols. There were concerns about their possible role of viral rebound and adverse events in COVID-19 time but results from a randomized clinical trial enrolling patients with COVID-19 indicated that the use of dexamethasone decreased mortality in hospitalized patients requiring supplemental oxygen or mechanical ventilation [10]. Despite these consequences, we should still consider decreasing the steroid exposure in the elderly population. Caution should be given to use of asparaginase regarding the increased risk of thrombotic complications of this drug and may mimic the COVID-19 related coagulopathies. The use of blinatumomab and inotuzumab (with ursodeoxycholic acid as prophylaxis) should not be deferred as their efficacy in terms of survival has been established in the frontline setting regardless of age [6]. It’s important to note that these agents also minimize myelosupression and risk of COVID-19. According to recommendations from a panel of international experts, treatment with anti-CD20 monoclonal antibodies should be delayed if possible due to hypogammaglobulinemia [9]. The decision to withhold these agents should be made only in patients in which the risk outweighs the benefit.

Consolidation with blinatumomab is considered if the patients are positive for measurable residual disease (MRD) after two cycles of chemotherapy. If the patients achieved MRD negativity, advancement to maintenance should be recommended [10]. The most of the ALL protocols include 2 years of maintenance therapy after induction/consolidation. As of March 19th 2020, GRAALL-14 investigators recommended to omit vincristine and prednisolone during maintenance, whilst continuing 6-mercaptopurine and methotrexate [11]. For patients in first CR, AHSCT can be postponed but the patients in second CR should undergo AHSCT promptly as the risks of relapse are high [12,13]. Inotuzumab could be given as the first salvage therapy instead of blinatumomab for administering outpatient treatment. It was also recommended to delay CD19 CAR T-cell therapies. For patients with relapsed or refractory ALL, each center should carefully evaluate the risks and benefits of pursuing a curative approach on a case-by-case basis [10].

#### 4.1.2. Ph-positive ALL

TKIs are the backbone of the treatment. Second generation TKI with reduced dose steroids are recommended to reduce the duration of hospital stay in the COVID-19 time. One has to keep in mind to continue administering a total of 12 intrathecal chemotherapies for central nervous system prophylaxis [6].

### 4.2. Hairy cell leukemia

Most centers are using cladribine as an initial treatment of patients with hairy cell leukemia. However, cladribine is immunosuppressive, it’s important to avoid severe neutropenia whenever possible to minimize the risk of acquisition of SARS-CoV-2 [14]. Antibiotic prophylaxis and G-CSF support are administered to shorten the period of neutropenia and reduce the risk of a secondary bacterial infection after viral infection.

### 4.3. Nonhodgkin lymphoma

#### 4.3.1. Aggressive B-cell lymphomas

The diffuse large B-cell lymphoma (DLBCL) is the most common aggressive lymphoma and majority of patients with DLBCL require prompt treatment. Treatment of newly diagnosed patients, often with intent to cure, has to be shifted to outpatient setting whenever feasible. The combination of rituximab with cyclophosphamide, doxorubicin, vincristine, and prednisone (R-CHOP) continues to be the standard of care for DLBCL therapy during the COVID-19 pandemic [15]. R-mini-CHOP with G-CSF support may be used for the elderly patients. Four cycles of R-CHOP rather than combined modality therapy is recommended for limited stage (stage I/II) DLBCL if PET-CT is negative at the end of treatment. None of the experts have yet advised to change treatment for those already receiving the treatment during COVID-19 pandemic [15]. It is acceptable to recommend hypofractionation or omitting consolidation RT to patients with bulky disease in those who are in complete metabolic response after chemotherapy as reflected in the International Lymphoma Radiation Oncology Group Emergency Guidelines [16].

The primary mediastinal lymphoma (PML) is a subgroup of aggressive lymphoma that has been reported excellent results with R-DA-EPOCH. However, there is no phase 3 randomized trial comparing R-CHOP and R-DA-EPOCH. Hence, R-CHOP followed by radiotherapy (RT) remains the standard therapy in the COVID-19 time [16]. Other aggressive lymphomas such as Burkitt’s lymphoma, plasmablastic lymphoma, and lymphoblastic lymphoma, the treatment strategy should not be changed based on clinical and resources factors during the COVID-19 pandemic. These are highly aggressive lymphomas that require prompt treatment despite of having high risk of life-threatening complications [15].

Most experts do not recommend postponement of chemotherapy in time of COVID-19 where the defer itself is likely to adversely impact on patient’s outcome since dose intensity and time are substantial [16]. Subcutaneous R should be advised to shorten patient time in health care facilities [15]. G-CSF support may be suggested to lighten neutropenia, and reduce the risk of febrile neutropenia. Telemedicine is highly encouraged at most centers and defer medical appointments for patients in CR or in patients in which no prompt change in therapy is expected.

In the relapse setting, most experts are suggesting outpatient regimens such as gemcitabine-based regimens (rituximab, gemcitabine, cisplatin, and dexamethasone (R-GDP)), or oxaliplatin-based (rituximab, dexamethasone, cytrabine, and oxaliplatin (R-DHAOX)) instead of R-DHAP (rituximab, dexamethasone, cytarabine, cisplatin) or R-ICE (rituximab, ifosfamide, carboplatin, etoposide) during COVID-19 pandemic [16]. The autologous stem-cell transplant (ASCT) should not be postponed in patients with chemosensitive disease or at least in partial response (PR) by CT scan. If there is no complete metabolic response on PET-CT after salvage therapy, the decision to proceed with ASCT should be considered individually by taking into consideration poorer outcome, especially in primary refractory disease [16,17]. Some institutions may need to delay ASCT due to lack of available ICU beds and blood shortages in COVID-19 time. Lenalidomide-based regimen or polatuzumab (approved in the third-line in the USA) with bendamustine may be recommended in the relapse setting but it is important to note that bendamustine dose reduction should be considered due to high rates of febrile neutropenia. In addition, off-label use of ibrutinib may be also considered [4].

Treatment of post-ASCT relapses are challenging in the COVID-19 time. CAR T-cell therapy and AHSCT are resource intensive and severe immunosuppressive and are likely to be less attainable [16,18].

#### 4.3.2. Mantle cell lymphoma

As in the pre-COVID era, indolent mantle cell lymphoma (MCL) with nonbulky disease and asymptomatic patients may undergo a watch-and-wait strategy, including symptom education and potentially surveillance imaging [16]. For young and fit patients in need of prompt treatment with aggressive MCL high-dose cytarabine-based induction therapy followed by ASCT and R maintenance may be considered. No consensus was achieved on R maintenance during the COVID-19 pandemic. If there is significant community transmission of COVID-19, R maintenance should be avoided due to increased risk of neutropenia and infection, without proven overall survival benefit [16,17]. Controversially, ASCT may need to be delayed in the setting of centers with the limited capacity during COVID-19 outbreak. If ASCT is decided to postpone, stem cell mobilization and collection should be performed after 3–4 cycles of induction therapy [16].

For elderly or unfit patients, bendamustine-rituximab (BR), R-CHOP or rituximab with cyclophosphamide, vincristine and prednisone (R-CVP) therapy with G-CSF support may be suggested [16]. The benefit of R maintenance after BR has not been clarified yet, therefore, so not recommended in the current pandemic, but an OS benefit with R maintenance after R-CHOP and after ASCT is well known [16]. Therefore, patients should be evaluated individually.

In the relapsed setting, the centers should choice regimens according to individual patient’s features and pandemic evaluations. Based on this, oral drugs and immunotherapy combinations may be preferred, such as Bruton’s tyrosine kinase (BTK) inhibitors and lenalidomide with R if not previously given. AHSCT should be postponed in stable patient [16].

#### 4.3.3. Indolent B-cell lymphomas

As in the pre-COVID-19 time, patients with indolent B-cell NHL do not require prompt initiation of therapy unless they have symptomatic nodal/extranodal disease, end-organ compromise or life-threating cytopenias [15]. If the indication is borderline, the initiation of planned treatment can be based on weighing risk-benefit ratio between patient and physician, taking into account disease features, in particular nonaggressive behavior of disease, personalized infection risk and patient choice. Therefore, a watch-and-wait approach might also be preferred for oligo-symptomatic patients in order to avoid immunosuppressive treatment during COVID-19 pandemic [15].

When treatment is indicated, protocols requiring the least immunocompromise and fewest clinic visits should be chosen during COVID-19 outbreak. For patients with symptomatic sites of disease, limited palliative RT (4 Gy in one to two fractions) should be considered with minimal toxicity. Many experts are advising R-CVP (or obinutuzumab-CVP for follicular lymphoma) or R-CHOP (or obinutuzumab-CHOP for follicular lymphoma) with G-CSF support and R maintenance. R monotherapy may also be recommended to frail patients during COVID-19 pandemic [16,17].

For patients who have already obtained an excellent response to chemoimmunotherapy, decreasing the number of treatment cycles may be suggested or a change of treatment to less immunosuppressive options in the time of COVID-19. It is reasonable to defer R maintenance to allow for faster B-cell recovery during the COVID-19 outbreak. Some experts have held R maintenance in elderly patients and in younger patients with low immunoglobulin (Ig) levels and suggest IVIG supplementation if available [16]. Management of relapsed/refractory indolent lymphoma should depend on constitutional symptoms and indications for treatment. Oral agents such as ibrutinib and lenalidomide with rituximab should be considered but bendamustine must be avoided because of its immunosuppressive properties [15,16].

#### 4.3.4. Chronic lymphocytic leukemia

Chronic lymphocytic leukemia (CLL) is usually a disease of the elderly population associated with impaired immunity even with early stage disease, and increased risk for COVID-19 and related complications. In patients not meeting International Workshop on chronic lymphocytic leukemia (IWCLL) indications for treatment, the watch-and-wait period should continue to be applied [19]. Therefore, medical appointments for patients may be postponed with a view to primary prevention by minimizing potential exposure. Telemedicine and local laboratories are highly encouraged during COVID-19 outbreak.

In patients in need of immediate therapy, the best treatment option should be based on individual factors including symptom burden and comorbidities, along with molecular and cytogenetic abnormalities [7]. If reasonable, initiation of new therapies to patients with CLL should be deferred to minimize the number of hospital visits and potential hospitalization in the presence of this pandemic. One has to keep in mind that treatment should be considered to delay in oligo-symptomatic patients, as well as patients with nonlife threatening cytopenias.

Fludarabine, cyclophosphamide, and rituximab (FCR) combination is the standard chemotherapy regimen in fit patients with CLL [20]. However, during COVID pandemic FCR may not be preferred due to significant immunosuppression, including grade 3/4 cytopenia and febrile neutropenia [21]. If the patient is already treated with FCR, early cessation of treatment may be considered after 3–4 cycles because of clinically significant treatment-related cytopenias or achieved disease control [20]. There was no consensus on using BR (bendamustine, rituximab) instead of FCR in patients in need of therapy in time of COVID-19. In addition, the use of monoclonal antibodies (rituximab and obinutuzumab) should be avoided or skipped if convenient, because of B-cell depletion which may potentially reduce the humoral response to virus [20,21].

The management of CLL treatment has moved into highly effective oral targeted therapies over the past few years. In the era of COVID-19 pandemic, we should consider to use the novel oral therapies, including ibrutinib, acalabrutinib, and venetoclax, both in the newly diagnosed and relapsed settings, especially in patients with high-risk cytogenetics (del17p and TP53 disruption)^8^. It is important to note that ibrutinib and acalabrutinib are generally well tolerated and limit the time spent in the clinic and resource utilization at the time of treatment initiation compared with the venetoclax and obinituzumab combination. When considering venetoclax in combination with anti-CD20 therapy, it is recommended to defer rituximab/obinutuzumab and administer venetoclax as monotherapy [21]. However, venetoclax requires tumour lysis syndrome (TLS) monitoring and leads to higher rates of FEN than treatment with BTK inhibitors. On the other hand, BTK inhibitors may cause atrial fibrillation and an increased risk of bleeding and one has to keep in mind that patients with severe COVID-19 often develop severe thrombocytopenia [10,11].

If the CLL patients are currently on an oral targeted treatment without any complications, it’s recommended to continue with the same therapy^7^ [6,10,11]. We have to emphasize that BTK inhibition may compromise innate immune response as well as T and B-cell functions resulting in a decreased cellular and humoral immune response to SARS-CoV-2 [22]. Treon and his colleagues had reported that BTK inhibition could lead to inhibition of cytokine production and potentially decrease the risk of hyperinflammation associated with COVID-19. Based on these results, ibrutinib and acalabrutinib are now being investigated for the treatment of cytokine release syndrome associated with COVID-19 [17].

In CLL patients, IVIG treatment is considered to continue during COVID-19 pandemic. But it is reasonable to suggest less frequent infusions (e.g., every 6–8 weeks) targeting an IgG level of 400–500 mg/dL. In addition, given the increased risk of thromboembolic events in the patients with COVID-19, it is recommended to weigh the risk and benefits for each patient and to monitor closely for thromboembolic symptoms^7^.

Currently, there is not enough evidence to recommend changing the specific classes of targeted CLL drugs in patients infected with SARS-CoV-2 and discontinuation or continuing treatment decisions should be made on case-by-case basis.

### 4.4. Hodgkin’s lymphoma

It is recommended not to delay the treatment of HL during COVID-19 outbreak [19]. However, since there is no clear consensus on the role of more intensive regimens that consume more resources in time of COVID-19, ABVD has been considered the good choice as initial treatment [15]. Of note, pulmonary toxicity associated with bleomycin may become a risk factor for those who acquire COVID-19 and also may mimic the symptoms of COVID-19 [16].

Regarding relapsed patients with HL, outpatient salvage chemotherapy protocols should be considered including gemcitabine-based regimens or novel agents such as brentuximab vedotin (BV) and checkpoint inhibitors (CPI) (nivolumab) [6,15,16]. In addition, it is not recommended to postpone the ASCT depending upon accessibility, due to the chance of cure [14]. There is no clear consideration for BV consolidation in the COVID-19 outbreak. A novel agent (BV or CPI) might be preferred in the second-line salvage setting or transplant-ineligible patients [15,16]^9^. But it is important to mention that the risk of pulmonary toxicity with CPI in the time of COVID-19 is unknown.

## 5. Multiple myeloma

The median age at diagnosis for myeloma patients is 69 years and elderly patients are more susceptible to have comorbidities compromising the immune system [23]. In particular, these patients have significant immune dysfunction and an uncontrolled malignant clone affecting the host immunity by suppressing normal B-cell development and function long before the MM diagnosis [24]. The overall tumor burden, as shown by laboratory and imaging studies, and the previous history of asymptomatic long-standing paraproteinemia should also be taken into consideration. One has to keep in mind that myeloma drugs such as immunomodulatory drugs (IMID) and proteasome inhibitors (PIs) also cause more immunosuppression and contribute more infections [25,26].

Prophylaxis against varicella-zoster virus and pneumocystis jirovecii are required for myeloma patients. Levofloxacin prophylaxis for the first three months of induction treatment is also highly recommended [27]. In addition, myeloma patients are at an increased risk for thromboembolism that may alter in accordance with patient, disease status, or treatment-related characteristics and may be aggravated with SARS-CoV-2 infection that promotes a hypercoagulation state as well [28]. Prophylactic anticoagulation should be given according to local or international guidelines and low-molecular-weight heparin (LMWH) may be taken into consideration for countries with high incidence of COVID-19 in myeloma patients under IMIDs regardless of their thrombotic risk [29].

### 5.1. Monoclonal gammopathy of undetermined significance and smoldering MM

There is no disagreement regarding the management of patients with monoclonal gammopathy of undetermined significance (MGUS) and standard-risk smoldering MM (SMM). These patients should be followed up long-term with no active intervention. For high-risk patients, encouraging results with lenalidomide was established but this has not change the clinical practice outside clinical trials in most countries [29,30]. We currently believe that including these patients in clinical trials is the best strategy but because of the current pandemic situation, many trials closed the new patient administration so we would advise close monitoring of these patients for development of symptomatic disease requiring therapy.

### 5.2. Newly diagnosed MM

According to the European Myeloma Network (EMN) recommendations, induction therapies should not be postponed for newly diagnosed MM (NDMM) patients with active disease. It was suggested to screen all patients for SARS-CoV-2 infection before initiating therapy as myeloma treatment can aggravate the adverse events of an active COVID-19. For myeloma patients testing positive for SARS-CoV-2, holding therapy was recommended until they have convalesced from acute disease [29]. Standard firstline treatment using bortezomib, cyclophosphamide, and dexamethasone (VCD) or bortezomib, lenalidomide, and dexamethasone (VRD) should be considered [24,29]. We want to point out that subcutaneously once a week bortezomib administration might be preferred rather than intravenously or twice a week. In addition, the dose of dexamethasone should be reduced to 20 mg weekly [23]. Oral drugs combinations (ixazomib, lenalidomide, and dexamethasone (IRd); cyclophosphamide, lenalidomide, and dexamethasone (CRd)) may also be used in NDMM patients to reduce the risk of weekly clinic visits and exposure [25,30]. It’s important to note that these regimens have not been compared head to head with known standard treatment options, but the response rates from early phase trials are comparable. In the maintenance setting, oral therapies which will reduce hospital visits should be preferred as much as possible.

#### 5.2.1. Transplant-eligible NDMM

In considering, the anticipated immunosuppression following ASCT, it is suggested to delay mobilization, stem cell harvest, conditioning and ASCT, particularly in patients with standard risk disease [28]. Physicians may also postpone ASCT in patients with marginal fitness due to age or comorbidities [29]. In case of close contact with a person infected with SARS-CoV-2, a deferral of the stem cell harvests and any transplant procedure is recommended until at least 14 days, and preferably 21 days from the last contact. In addition, patients should be closely monitored for the presence of COVID-19 with confirmed PCR negativity before transplant procedure is undertaken^3^.

In myeloma patients diagnosed with COVID-19, ASCT should be delayed until the patient is asymptomatic and has two negative PCR swabs taken at least 24 h apart. In patients with moderate to severe COVID-19, it can be considered to allow enough time for the lung function and general performance to reach to pre-COVID-19 values^3^.

Patients who are stable on maintenance with no major side effects should continue on their treatment. If the patient is on dexamethasone, tapering it down may be considered. In addition, these patients should visit the clinic every 3 months [28]. Monitoring should be planned in the closest laboratory and phone visits may be used for toxicity control.

#### 5.2.2. Transplant-ineligible NDMM

All-oral regimens (e.g. lenalidomide with dexamethasone (Rd)) should be considered in order to minimize hospital visits. Dexamethasone can be reduced to 20 mg weekly and even de-escalation (or even interruption) should be considered for responding myeloma patients [24,29,32].

#### 5.2.3. Relapsed/refractory disease

Depending on the COVID-19 prevalence and available clinical resources in the community, monthly close monitoring may be advised for biochemical relapses, especially for patients with a slow increase in the paraprotein level [29]. Otherwise, patients with refractory disease, new onset of CRAB features (hypercalcemia, renal failure, anemia, and bone disease) or those with a biochemical relapse and a history of aggressive relapse with impairment of the clinical presentation will require next-line, immediate treatment. Regarding the selection of treatment protocol, orally given agents should be advised [29].

Myeloma patients diagnosed with COVID-19 should be treated as per standard guidelines starting from isolation measures. Asymptomatic patients for COVID-19 should be quarantined at home for at least 14 days, under close surveillance for determining COVID-19-associated signs and symptoms, in such cases antimyeloma therapy should be deferred [28]. In patients with CRAB signs or other aggressive myeloma features, treatment should not be delayed [24,29]. One has to keep in mind that steroids and/or other drugs inducing lymphopenia should be discontinued.

In the event of emergence of symptomatic infection, treatment might be hold and steroids should be tapered down with the goal of discontinuation until full recovery from COVID-19 [24,29]. For patients enrolled in a clinical trial, investigational agents should be withdrawn until COVID-19 resolution and the reporting should be done with the corresponding guidelines. In general, COVID-19 infection is reported as an adverse event of special interest unless it fulfills the well-established criteria for a serious adverse event [29].

Myeloma patients are administered several drugs for supportive care in addition to drugs with direct antimyeloma activity. Caution should be given to polypharmacy. Many agents that are being evaluated against SARS-CoV-2 (hydroxychloroquine, azithromycin and remdesivir) may have significant interactions with other drugs and may result in significant hepatic, cardiac or renal toxicity [33,34,35]. Hence, close monitoring of organ functions is important.

## 6. Conclusions

The COVID-19 pandemic represents a tremendous challenge and has been an unusual historic pandemic of recent times. This pandemic created an unprecedented medical, logistical, financial, and public health hurdles to the delivery of optimal care for patients with hematologic malignancies. Because of either the primary malignancy or the severe myelosuppression and lymphodepletion due to chemotherapy regimens, patients with hematologic malignancies have an increased risk of SARS-CoV-2 infection and severe COVID-19 disease [11]. Curative therapy for many patients with hematologic malignancies includes a dose intensive chemo-radiation protocols followed by ASCT or AHSCT [36]. Therefore, COVID-19 pandemic is currently an important challenge in the setting of treatment of hemato-oncological malignancies [37]. The technologic explosion of telemedicine and home care platforms are highly encouraged at most centers and defer medical appointments for patients in CR or in patients in which no prompt change in therapy is expected. While routine elective procedures have been postponed for a while, scheduling and performance of the diagnostic tests, staging, and chemotherapy clearance procedures have continued [11]. In some cases, what was once thought to be impossible has become a normal routine. COVID-testing platforms have been spread remarkably. Clinical trial operations have remained open, if scaled down, in many institutions during the pandemic, and study logistics, including consenting, sample acquisition and transportation as well as study visits have been made more flexible. While the peak of the pandemic may soon be gone with the use of vaccine, the risk of impending additional waves of infection remains, and our level of alertness and the operational response to future challenges need to be pursued.

## Footnotes

1 Website: https://www.worldometers.info/coronavirus [accessed 18 November 2020].

2 American Society of Hematology (2020). Coronavirus Disease (COVID-19) and Acute Myeloid Leukemia: frequently asked questions [online]. Website: https://www.hematology.org/covid-19/covid-19-and-acute-myeloid-leukemia [accessed 18 November 2020].

3 European Hematology Association (2020). Recommendations for specific hematologic malignancies, AML and COVID-19 pandemic [online]. Website: https://ehaweb.org/covid-19/covid-19recommendations/recommendations-for-specific-hematologic-malignancies/ [accessed November 2020].

4 European Society for Blood and Marrow Transplantation (2020). Coronovirus disease COVID-19 Recommendations [online]. Website: version 12-December 2, 2020. https://www.ebmt.org/sites/default/files/2020-12/EBMT%20COVID-19%20guidelines%20and%20summary%20v.13.pdf [accessed 2 December 2020].

5 American Society of Hematology (2020). Coronavirus Disease (COVID-19) and myeloproliferative neoplasms: frequently asked questions [online]. Website https://www.hematology.org/covid-19/covid-19-and-myeloproliferative-neoplasms. [accessed 14 November 2020].

6 American Society of Hematology (2020). Coronavirus Disease (COVID-19) and myeloproliferative neoplasms: frequently asked questions [online]. Website https://www.hematology.org/covid-19/covid-19-and-myeloproliferative-neoplasms. [accessed 14 November 2020].

7 American Society of Hematology (2020). Coronavirus Disease (COVID-19) and chronic myeloid leukemia: frequently asked questions [online]. Website https://www.hematology.org/covid-19/covid-19-and-cml [accessed 25 September 2020].

8 American Society of Hematology (2020). Coronavirus Disease (COVID-19) and chronic lymphocytic leukemia: frequently asked questions [online]. Website https://www.hematology.org/covid-19/covid-19-and-cll [accessed 17 November 2020].

9 Website: https://www.hematology.org/covid-19/covid-19-and-hodgkin-lymphoma. [accessed 10 December 2020].
